# A Rare Presentation of Crohn’s Disease: A Case Report

**DOI:** 10.7759/cureus.99101

**Published:** 2025-12-13

**Authors:** Inês Marques Ferreira, Rui Coelho, Célia Cruz, Inês Ferreira Henriques, Sofia Ribeiro

**Affiliations:** 1 Internal Medicine, Unidade Local de Saúde de Santo António, Porto, PRT; 2 Internal Medicine, Centro Hospitalar Universitário do Porto, Porto, PRT; 3 Home Hospitalization Unit, Unidade Local de Saúde de Santo António, Porto, PRT

**Keywords:** crohn’s disease (cd), extraintestinal, extraintestinal manifestation of inflammatory bowel disease, inflammatory bowel disease, malabsortion, pyogenic liver abscess (pla)

## Abstract

Crohn’s disease (CD) is a chronic, transmural inflammatory disorder that may affect any segment of the gastrointestinal tract and is frequently associated with extraintestinal manifestations, including hepatobiliary complications. Pyogenic liver abscesses (PLAs) represent a rare extraintestinal manifestation and are exceptionally uncommon as the initial presentation of CD, with only a few cases reported to date. We describe a previously healthy 39-year-old man, an active smoker, presenting with five days of fever, shivering, myalgias, anorexia, and constipation. On admission, he was febrile, tachycardic, underweight, and had hepatomegaly with diffuse abdominal tenderness. Laboratory studies revealed leukocytosis, microcytic anemia, elevated inflammatory markers, mild cholestatic changes, and deficiencies in folic acid and vitamin D, suggesting malnutrition. Serologies for hepatitis B and C and HIV, as well as autoimmune liver panels, were negative. Abdominal ultrasound and subsequent CT imaging identified four liver abscesses, the largest measuring 5.8 cm, and terminal ileum wall thickening with reactive mesenteric lymphadenopathy. Blood and aspirate cultures grew *Streptococcus intermedius*, prompting tailored antibiotic therapy and percutaneous drainage. Given the absence of known abdominal pathology, colonoscopy was performed and revealed marked ileocecal congestion and ulceration. Although biopsies showed nonspecific inflammatory activity without granulomas, elevated fecal calprotectin, positive anti-*Saccharomyces cerevisiae* antibodies, and imaging characteristics consistent with skip lesions strongly supported the diagnosis of CD. Because the active infection precluded initiation of immunosuppressive therapy, the patient underwent exclusive enteral nutrition and smoke cessation with gradual resolution of the abscesses under targeted antibiotics. Once the infection cleared, ustekinumab was initiated. At three months, the patient demonstrated weight recovery and clinical remission and has since remained asymptomatic. PLA is markedly more frequent in patients with CD than in the general population, although its appearance as the initial disease manifestation is rare. This case adds to the limited number of reported CD-associated PLAs and is notable for the absence of preceding intestinal symptoms. Early etiological investigation of PLA, especially in young patients without comorbidities, is essential for timely diagnosis and management. Moreover, the coordinated care provided through a Home Hospitalization Unit contributed significantly to treatment adherence and improved prognosis.

## Introduction

Crohn’s disease (CD) is a chronic inflammatory disorder that most commonly affects the terminal ileum but can involve any segment of the gastrointestinal tract. The disease is characterized by transmural inflammation and a discontinuous distribution of affected areas, known as “skip lesions” [1]. As a systemic illness, it can also present with extraintestinal manifestations, reported in more than one-third of patients [2], including liver and biliary tract complications, namely, cholelithiasis and primary sclerosing cholangitis.

Pyogenic liver abscess (PLA) is a rare extraintestinal manifestation, usually developing secondary to biliary infection, by direct contiguity or through infections of the organs drained by the portal vein, like in appendicitis or diverticulitis. As the initial manifestation of CD, PLA is even rarer, having been described only eight times between 1988 and 2022 [3-9].

Diabetes mellitus, endoscopic biliary drainage, abdominal surgery, long-term steroid therapy, fistulizing disease, and malnutrition are all likely predisposing factors [2]. Most cases of PLA are seen in young patients with long-standing and uncontrolled CD, as persistent transmural inflammation can further facilitate the invasion of the liver by bacteria [10]. 

We report a patient who presented with multiple liver abscesses as the initial manifestation of CD.

This article was previously presented as a poster at the 2º Congresso Nacional de Hospitalização Domiciliária on June 4, 2022.

## Case presentation

A 39-year-old Caucasian male, active smoker (eight pack-years), without any relevant past medical history, presented with five days of fever, shivering, and myalgias. In addition, he reported anorexia and constipation for the past three days, without abdominal pain. 

On admission, of note, the patient was febrile, hemodynamically stable, tachycardic, with a respiratory rate of 32 cycles per minute and no respiratory failure; he had a sick appearance, with low weight (weight 62 kg, body mass index of 18.7 kg/m^2^) and a palpable liver 1-2 cm below the costal grid in the midclavicular line, with blunt borders and global abdominal tenderness on palpation.

Laboratory analysis was significant for leukocytosis with left shift, microcytic anemia, mild thrombocytosis, and elevated gamma-glutamyl transferase (2.5 times above normal), but with normal transaminases and bilirubin. C-reactive protein was increased (Table 1).

**Table 1:** Analytical data upon admission in the emergency department. MCH: mean corpuscular hemoglobina; MCHC: mean corpuscular hemoglobin concentration; MCV: mean corpuscular volume; fL: femtolite; pg: picograms

	Value	Reference range
Hemoglobin (g/dL)	11.3	13-17
Hematocrit(%)	33.6	40-50
MCV(fL)	77.2	83-101
MCH(pg	26	27-32
MCHC(g/dL)	33.6	31.5-34.5
White blood cell (x10^3^/mL)	21.89	4-11
Neutrophils (x10^3^/mL)	18.95	2.00-7.5
Lymphocytes (x10^3^/mL)	0.96	1.50-4.0
Monocytes (x10^3^/mL)	1.77	0.20-0.80
Eosinophils (x10^3^/mL)	0.00	0.04-0.40
Basophils (x10^3^/mL)	0.07	0.02-0.10
Platelet count (x10^3^/mL)	415	150-400
Creatinine (mg/dL)	0.99	0.7-1.2
Urea (mg/dL)	26	10-50
Total bilirubin (mg/dL)	1.26	0.2-1.0
Direct bilirubin (mg/dL)	0.57	0.0-0.3
Indirect bilirubin (mg/dL)	0.69	0.0-1.0
Alanine aminotransferase (U/L)	58	10-44
Aspartate aminotransferase (U/L)	40	10-34
Alkaline phosphatase (U/L)	147	40-129
Gamma-glutamyl transpeptidase (U/L)	165	10-66
C-reactive protein (mg/L)	178.65	0.0-5.0

The iron study was compatible with inflammatory anemia, and folic acid and vitamin D levels were low, suggesting a malabsorptive syndrome.

Serologies for hepatitis B and C and HIV were negative. The autoantibody panel for hepatic autoimmune diseases also came back negative.

The abdominal ultrasound showed an enlarged liver with a 18.6 cm bipolar diameter in the mid-clavicular line, with at least four solids, slightly hyperechogenic focal lesions, with the two largest located at the VII/VII transition (measuring 4.1 cm) and at the high convexity of segment IV (measuring 4.7cm), showing a hypoechoic halo.

Blood cultures were collected, and the patient was started on empiric antibiotic therapy with ciprofloxacin and admitted to the hospital for study of the hepatic lesions.

During the first days of hospitalization, the fever and pain persisted despite antibiotics. On the fifth day of admission, blood cultures returned positive for Streptococcus intermedius, a member of the *Streptococcus milleri *group, which is susceptible to penicillin, ampicillin, and vancomycin. The antibiotic therapy was adjusted to the susceptibility test and switched to vancomycin. 

**Figure 1:**
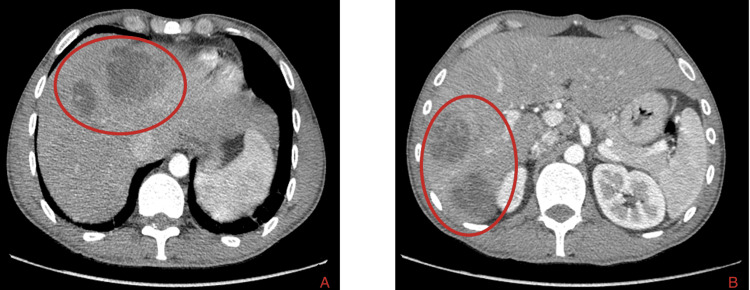
Computerized tomography of the abdomen and pelvis demonstrating multiple liver abscesses (red circle). In Figures A and B, identified by the red circles, there are four hepatic abscesses located in segments V–II, VIII, V, and VI.

**Figure 2:**
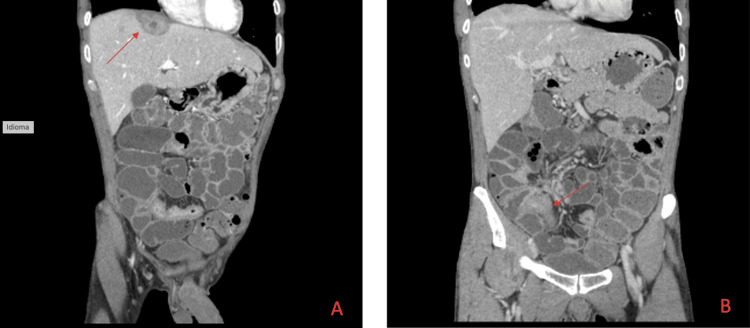
Abdominal Entero CT images of the pyogenic abscess and inflammation of the ileocecal valve. A: One pyogenic liver abscesses in the process of being resolved, identified by the red arrow. B: Diffuse segmental parietal enhancement of the terminal ileum, involving the ileocecal valve, identified by the red arrow.

A computerized tomography (CT) scan was performed, which revealed hepatomegaly (22 cm) and four images with imagiological characteristics compatible with liver abscesses in segments IV-II (5.8cm), segment VIII (3.7cm), and segments V and VI, measuring 4.4cm and 4.7cm, respectively (Figures 1, 2). At the same time, parietal thickening of the terminal ileum was observed, indicating ileitis, with multiple reactive adenomegalies in the root of the mesentery. 

Metronidazole was added to the antibiotic strategy at this point. The patient underwent an ultrasound-guided aspiration of the biggest abscess, draining six cc of pus from the VI segment, with fluid cultures also coming back positive for Streptococcus intermedius.

The presence of liver abscesses with cocci of this group, without known abdominal pathology, may be indicative of an occult gastrointestinal lesion. The patient underwent colonoscopy with progression to the ileocecal valve, which showed marked congestion and a clean-based ulcer. Biopsies came back negative for Koch bacilli, and pathology showed moderate inflammatory activity without granulomatous lesions. 

Probable CD was assumed, with severe extraintestinal manifestation and moderate disease activity (Crohn Disease Activity Index 247 points). Elevated calprotectin and positive anti-*Saccharomyces cerevisiae* antibodies, with remaining negative serological and immunological studies and malabsorptive syndrome (anemia, folic acid, and vitamin D deficiency).

Since it was not possible to transpose the ileocecal valve due to congestion, a CT with small bowel enterography was performed, describing diffuse segmental parietal thickening of the terminal ileum, involving the ileocecal valve, with inflammatory enhancement of the mucosa and luminal sub-stenosis; another two segments with similar characteristics were observed, in the distal ileum and in the right iliac fossa, compatible with skip lesions, highly suggestive of CD.

The diagnosis of CD was made, and the patient was started on exclusive enteric nutrition as an attempt to induce remission until the infectious condition resolved. Smoking cessation treatment was also started, with good adherence. 

After one month of targeted antibiotics, the patient was switched to ceftriaxone, based on the antibiotic sensitivity test. A new CT was done, which showed a decrease in the size of all liver abscesses. He was discharged and scheduled for an appointment with a Digestive Diseases specialist, where he started ustekinumab, an anti-IL12/23 monoclonal antibody. After three months of therapy, the patient was already under culinary nutrition with weight recovery (BMI 22 kg/m^2^) and clinical remission. 

At present, the patient is still in clinical remission, without any flares since the diagnosis. 

## Discussion

PLA is an uncommon but clinically significant extraintestinal manifestation of CD. Although hepatobiliary complications are recognized in more than one-third of patients with inflammatory bowel disease [2], PLA remains rare and is typically linked to biliary tract disease, contiguous spread, or portal venous seeding from intra-abdominal infections such as appendicitis or diverticulitis [3]. Despite this, its occurrence in CD is estimated to be 10-15 times higher than in the general population [1].

What distinguishes the present case is that the liver abscesses represented the initial manifestation of CD, an exceedingly rare scenario, with only a handful of similar cases described since 1988 [4-8]. Previous reports have shown that PLA in this setting frequently occurs in individuals with established CD, in whom transmural inflammation facilitates bacterial translocation and hepatic invasion [9]. Known predisposing factors, including immunosuppressive therapy, diabetes, and prior abdominal surgery, were absent in this patient [2], emphasizing the atypical nature of this presentation.

The isolation of *Streptococcus intermedius*, a member of the *Streptococcus anginosus* group, in both blood and abscess cultures was clinically meaningful. This organism is strongly associated with abscess formation, particularly of gastrointestinal origin, and its presence in liver abscesses should prompt investigation for an occult gastrointestinal source [10]. In this case, the microbiological findings were pivotal in directing further evaluation, ultimately revealing significant ileal inflammation.

Diagnosing CD relies on a combination of clinical, laboratory, endoscopic, and imaging findings. Elevated fecal calprotectin, positive anti-*Saccharomyces cerevisiae* antibodies, malabsorptive laboratory markers, and CT enterography demonstrating skip lesions collectively supported the diagnosis despite inconclusive initial histology.

Management of this patient underscores the importance of a sequential therapeutic strategy. Because immunosuppressive therapy is contraindicated in the setting of active infection, treatment began with targeted antibiotic therapy and percutaneous drainage, followed by exclusive enteral nutrition to induce remission while controlling systemic inflammation. Nutritional therapy, smoking cessation, and the support of a home hospitalization team were crucial for maintaining disease stability until biological therapy could safely be introduced. After resolution of infection, ustekinumab, a monoclonal antibody targeting IL-12/23, was initiated with excellent clinical response [11].

## Conclusions

PLA in CD carries a reported mortality largely due to delayed diagnosis and the severity of underlying disease. The present case contributes to the limited literature documenting PLA as the first presentation of CD and is particularly unique because the patient had no prior history of intestinal manifestations of the disease. 

The presence of an active serious infection made it impossible to introduce immunosuppressants, leading to the need to completely change the patient's eating habits and the lifestyle of the entire family. The role of the Home Hospitalization Unit team and its proximity care was vital for the treatment compliance, undoubtedly improving the patient's prognosis.

The authors would like to highlight the importance of a thorough etiological investigation of PLA, especially in young and otherwise healthy individuals, in whom a probable cause is not readily evident.
